# ncRNAs-Mediated Pyroptosis in Cerebral Ischemia-Reperfusion Injury: Pathophysiology, Mechanisms, and Therapeutic Perspectives

**DOI:** 10.3390/cimb47030141

**Published:** 2025-02-22

**Authors:** Ruiyi Xu, Quan Peng, Wen Chen, Xihua Cheng, Guozuo Wang

**Affiliations:** 1 Key Laboratory of Vascular Biology and Translational Medicine, Medical School, Hunan University of Chinese Medicine, Changsha 410208, China; 20233943@stu.hnucm.edu.cn (R.X.); chenwen@biochen.org (W.C.); 2 Key Laboratory of Hunan Province for Integrated Traditional Chinese and Western Medicine on Prevention and Treatment of Cardio-Cerebral Diseases, College of Integrated Traditional Chinese Medicine and Western Medicine, Hunan University of Chinese Medicine, Changsha 410208, China; pengquan@stu.hnucm.edu.cn

**Keywords:** cerebral ischemia-reperfusion injury (CIRI), pyroptosis, ncRNAs

## Abstract

Cerebral ischemia-reperfusion injury (CIRI) is a complex pathological process triggered by transient obstruction of blood flow and subsequent reperfusion, ultimately leading to intracellular disturbances such as oxidative stress, inflammatory responses, and programmed cell death. Among the various types of cell death, pyroptosis (an inflammatory kind of regulated cell death) has received increasing attention due to its involvement in key neurovascular unit cells, including endothelial cells, neurons, microglia, and astrocytes. Intriguingly, accumulating evidence demonstrates that non-coding RNAs (ncRNAs), including long non-coding RNAs, microRNAs, and circular RNAs, can modulate multiple stages of pyroptosis in CIRI. This review synthesizes recent findings on the ncRNAs-regulated pyroptosis in CIRI. We highlight the molecular underpinnings of pyroptotic activation following ischemic injury and discuss how ncRNAs shape these mechanisms. By elucidating the interactions between ncRNAs and pyroptosis-related pathways, we intend to present innovative viewpoints for early diagnosis and the development of potential therapeutic strategies to mitigate CIRI.

## 1. Introduction

Stroke is one of the most severe cerebrovascular diseases in the elderly, characterized by elevated rates of disability and death [1]. Clinically, stroke is classified into ischemic and hemorrhagic types. According to surveys, ischemic stroke accounted for 86.8% of all strokes in 2020 [2]. Ischemic stroke can lead to irreversible neuronal functional damage in brain tissue [3]. Subsequently, reperfusion can lead to secondary damage in ischemic brain tissue, known as CIRI [4]. CIRI is believed to be the primary mechanism of pathological injury in stroke. Moreover, various pathological processes, including inflammatory response [5], oxidative stress [6], autophagy [7], ferroptosis [8], mitochondrial dysfunction [9], angiogenesis [10], and others are closely related to CIRI, with the inflammatory response showing predominance.

Pyroptosis is a form of cell death that promotes inflammation. It is characterized by the formation of pores in the plasma membrane due to the action of gasdermin(GSDM) family proteins. Activation of cysteine aspartate-specific proteases (caspases) triggers their interaction with the GSDM protein family to create channels in the plasma membrane, leading to the discharge of pro-inflammatory substances, including interleukin-1β (IL-1β) and interleukin-18 (IL-18) [11]. Pyroptosis has emerged as a new area of research in CIRI [12], with abnormal pyroptosis reported in endothelial cells [13], neurons [14], microglia [15], and astrocytes in CIRI [16].

Many upstream signals cause pyroptosis, and ncRNAs have recently been found to be closely related to pyroptosis [17]. ncRNAs belong to the RNA class that do not encode proteins but play an essential role in regulating the expression of coding genes and their corresponding functions. Based on their length and shape, ncRNAs are classified into different categories. ncRNAs are primarily categorized into three subtypes based on structural features: (1) long non-coding RNAs (lncRNAs) (>200 nucleotides), (2) microRNAs (miRNAs) (20–22 nucleotides), and (3) circular RNAs (circRNAs), distinguished by their covalently closed circular topology. miRNAs can bind to complementary sequences in target mRNAs, leading to their degradation via the RNA-induced silencing complex (RISC) [18]. In contrast, lncRNAs and circRNAs regulate gene expression through various mechanisms, such as folding into specific structures to facilitate interactions with DNA, RNA, and proteins [19] or acting as miRNA sponges to prevent the degradation of target RNAs [20]. Studies have shown that ncRNAs are closely associated with and play a pivotal role in the occurrence and progression of CIRI [21].

In this review, we used “ncRNA”, “pyroptosis”, and “cerebral ischemia-reperfusion injury” as the core keywords and included their synonyms or related terms. We retrieved relevant articles between 1992 and 2024 from databases such as Web of Science and PubMed, which laid the foundation for this review. This article summarizes the research progress on ncRNAs-mediated pyroptosis in CIRI and provides new insights into the occurrence, development, diagnosis, and treatment.

## 2. The Role of Pyroptosis in CIRI

### 2.1. The Concept of Pyroptosis

In the early 1990s, researchers discovered a novel type of cell death in Shigella flexneri-infected macrophages [22]. In 2001, Brad Cookson and Molly Brennan named this newly discovered pro-inflammatory programmed cell death “pyroptosis” [23], defining it as a cell-death model associated with the release of pro-inflammatory factors [24]. From this point, pyroptosis was formally differentiated from apoptosis and other forms of cellular death mechanisms. Further research has shown that pyroptosis affects not only macrophages but also other cell types, including neurons, microglia, and endothelial cells. Moreover, pyroptosis has been linked to ischemia-reperfusion injury in vital organs such as the brain, heart, and liver [25,26,27].

**Figure 1:**
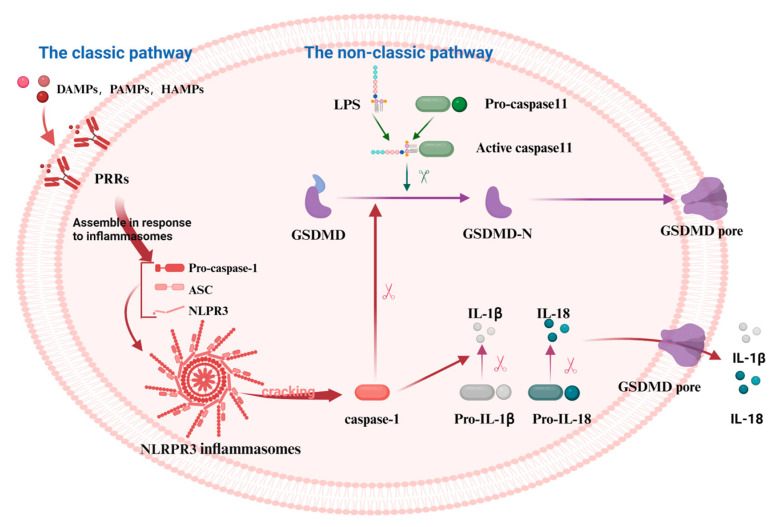
The mechanism of the classical and the non-classical pyroptosis pathway. In the classical pathway, PRRs recognize DAMPs, PAMPs, and HAMPs, activating the assembly of inflammasomes and pro-caspase-1. This leads to the cleavage of pro-caspase-1 into active caspase-1, which subsequently cleaves GSDMD, producing active GSDMD-NT that form cell membrane pores, resulting in pyroptosis. In the non-classical pathway, caspase-11 directly recognizes the lipopolysaccharide (LPS) of the pathogen, thereby activating the pyroptosis process via the non-inflammasome pathway. Caspase-11 binds to LPS and directly cleaves GSDMD, producing GSDMD-NT that form cell membrane pores, leading to pyroptosis. The figure was created using BioRender.

Pyroptosis is a programmed cell death (PCD) mode mediated by the GSDM protein family. It can be classified into the classic pathway, the non-classic pathway, and the alternative pathways [28]. NOD-like receptor thermal protein domain-associated protein 3 (NLRP3), a protein involved in immune responses, gets activated by various signals. These include damage-associated molecular patterns (DAMPs), pathogen-associated molecular patterns (PAMPs), and homeostasis-altering molecular processes (HAMPs). This activation in the classic pathway leads to the conversion of pro-caspase-1 into its active form, caspase-1. Furthermore, caspase-1 cleaves GSDMD, pro-IL-1β, and pro-IL-18 into their mature forms: the GSDMD-N-terminal fragments (GSDMD-NT), IL-1β, and IL-18 [29]. IL-1β and IL-18 stimulate the generation of additional inflammatory molecules, including cytokines, chemokines, and adhesion factors. Conversely, the NT is lipophilic and can move to the membrane film structure, where it gradually polymerizes to form pores, causing osmotic imbalance and rapid cell expansion, resulting in membrane rupture and leading to the leakage of cell contents [30]. However, no inflammasomes and cytoplasmic pattern recognition receptors (PRRs) are involved in the non-classic pathway, instead, cell death is mediated by the activation of other subunits in the caspase family (Figure 1). Rapid oligomerization of murine caspase-11 and human caspase-4/5 was found to lead to the cleavage of GSDMD, which forms membrane pores when stimulated by molecules such as LPS [31,32]. In addition to GSDMD, the cleavage of other members of the gasdermin family (GSDMA, GSDMB, GSDMC, and GSDME) results in the release of pro-inflammatory mediators such as IL-1β and subsequent cell lysis [33]. Recent studies have shown that caspase-3 can cleave GSDME to generate the GSDME-NT, causing pyroptosis [33,34]. Similarly, caspase-8 can regulate pyroptosis by cleaving GSDME and GSDMD proteins [35,36]. Under hypoxia, phosphorylated STAT3 (p-STAT3) binds to programmed death-ligand 1 (PD-L1), promoting PD-L1 nuclear translocation. This interaction activates GSDMC and caspase-8, thereby triggering the non-classic pyroptosis pathway [37].

### 2.2. The Role of Pyroptosis in CIRI

Neuroinflammation is generally considered an important secondary injury after stroke [5]. To date, inflammasomes NLRP1, NLRP3, NLRP6, NLRC4, and AIM have been associated with ischemic stroke [38,39,40]. According to statistics, the NLRP3 inflammasome is currently the most studied, and caspase-1 is activated in the sterile inflammatory response [41]. Findings demonstrate that pharmacological inhibition or genetic ablation of the NLRP3 inflammasome can enhance neurological performance [16]. The NLRP3 inflammasome is intimately linked to pyroptosis, a pro-inflammatory form of regulated cell death. In CIRI, neurovascular unit cells, including endothelial cells [42], neurons [43], microglia [44], and astrocytes [45], all undergo NLRP3-mediated pyroptosis, exacerbating neuroinflammation and tissue damage.

Pyroptotic endothelial cells release inflammatory cytokines, leading to vascular inflammation and blood-brain barrier disruption in CIRI [46]. A previous study investigating oxygen-glucose deprivation/reoxygenation (OGD/R) in endothelial cells and middle cerebral artery blockage/restoration (MCAO/R) mice revealed that NLRP3 is closely associated with pyroptosis. Endothelial cell pyroptosis is a crucial pathophysiological mechanism of ischemic stroke. Sodium Danshensu (SDSS) was found to inhibit the triggering of the NLRP3 inflammasome and reduce chloride efflux by binding to chloride intracellular channel 4 (CLIC4), thereby inhibiting caspase-1 cleavage and GSDMD-N shearing, suppresses the production and release of IL-1β and IL-18, reducing vascular endothelial cell pyroptosis, and alleviating CIRI [13]. Therefore, inhibiting the pyroptosis of vascular endothelial cells represents a potential therapeutic option for CIRI [47].

Pyroptotic neurons release inflammatory cytokines, leading to neuronal death in CIRI [48,49]. In MCAO mouse neurons and OGD/R neurons, BRCA1/BRCA2 Containing Complex Subunit 3 (BRCC3) was highly expressed, which activated the NLRP6 inflammasome, leading to pyroptosis [49]. Experiments showed that siRNA BRCC3 significantly downregulated the protein levels of NLRP6, cleavated caspase-1 and IL-1β, reduced neutrophil infiltration, and improved neurological dysfunction in MCAO mice [48].

Microglia polarize towards the pro-inflammatory (M1) phenotype, releasing inflammatory factors that lead to neuronal death and induce the activation of astrocytes into the A1 phenotype in CIRI. Microglia are crucial intrinsic immune cells that participate in regulating the body’s immune response and brain function [50]. Microglia can be divided into two types: the pro-inflammatory microglia (M1) and the anti-inflammatory microglia (M2) phenotype [51]. The M1 phenotype produces substances such as tumor necrosis factor-alpha (TNF-α) and inducible nitric oxide synthase (iNOS), which have toxic effects on nerves, exacerbating the inflammatory response and leading to further brain damage. In contrast, the M2 microglia exerts anti-inflammatory effects [52]. In a rat MCAO/R model, the M1 phenotype was activated by recognition of PAMPs or DAMPs, which enhanced NLRP3 and pro-IL-1β synthesis, promoting the maturation of GSDMD-NT and IL-1β, and leading to microglial pyroptosis. Consequently, inflammatory factors were released, leading to neuronal death and inducing the activation of astrocytes into the A1 phenotype. Therefore, inhibition of the NLRP3 inflammasome can alleviate pyroptosis and play a protective role in neurons [53,54,55,56].

Activated astrocytes exacerbate inflammatory reactions, leading to neuronal death in CIRI [57]. Scientific studies increasingly show a causal link between CIRI pathogenesis and astrocytic pyroptosis [58]. For example, pioglitazone can inhibit pyroptosis of astrocytes in brain damage [59], by regulating the expression of the NLRP3 inflammasome. Hispidulin inhibits astrocyte pyroptosis, alleviates neurological symptoms, reduces inflammatory responses, and improves cerebral edema [60]. Moreover, Lipocalin-2mediated astrocytic pyroptosis causes neuronal damage [16].

**Figure 2:**
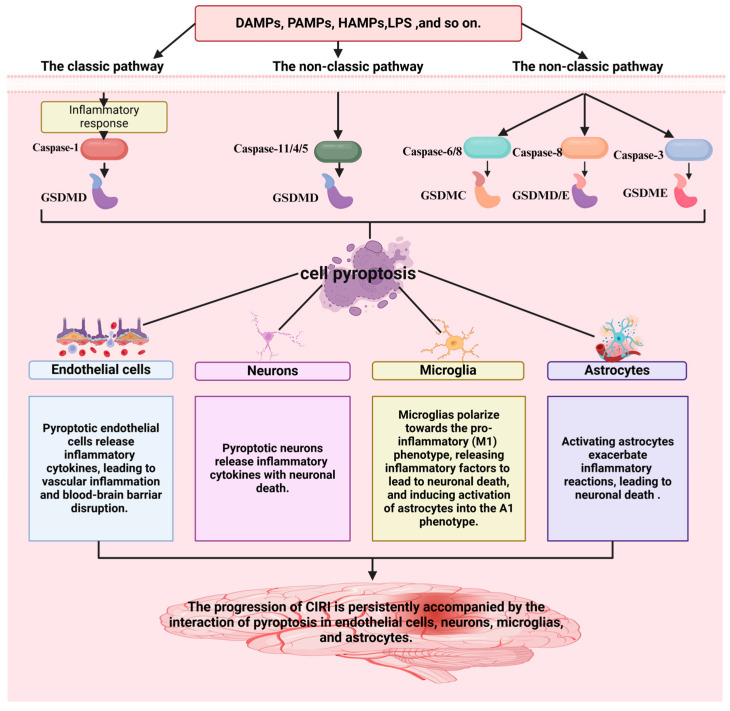
A strong association is observed between pyroptosis and the progression of CIRI. The figure was created using BioRender.

The above findings demonstrate that the progression of CIRI is persistently accompanied by the interaction of pyroptosis in endothelial cells, neurons, microglia, and astrocytes (Figure 2). Hence, inhibiting the pyroptosis of key cells (endothelial cells [42], neurons [43], microglia [44], and astrocytes [45]) in the neurovascular unit represents a potential therapeutic option for CIRI.

## 3. The Role of ncRNAs in CIRI

### 3.1. ncRNAs

RNA was once thought to be primarily a messenger, carrying instructions encoded by DNA so that other molecules, such as ribosomes, could use this code to produce proteins. However, in recent studies, researchers have discovered the existence of various types of RNA, the most important of which is ncRNA (mainly including lncRNA, miRNA, and circRNA), which does not participate in protein production. With the progress of research, the discovery of tens of thousands of ncRNAs has completely transformed this field and changed scientists’ views on physiology and disease development. ncRNA accounts for over 90% of the RNA in the human genome, yet most of it remains unexplored [61,62].

Specifically, miRNAs are involved in the post-transcriptional control of gene expression by binding to non-coding sequences located at the 3′-untranslated region (3′-UTR) of mRNA [63]. Precursor RNAs (pre-miRNAs) are transcribed and processed in the nucleus by RNA polymerase II, Drosha, and DiGeorge Critical Region 8(DGCR8) and then are exported from the nucleus via exportin-5 [64]. The pre-miRNA is further processed by Dicer and TAR RNA-binding proteins (TRBPs) to generate mature miRNAs that bind to Argonaute proteins to form RISC. RISC binds to mRNA through complementary miRNA response elements (MREs) that suppresses translation or induces the degradation of the target mRNA [65]. However, the pairing between miRNAs and target mRNAs may not be specific, allowing miRNAs to modulate a wide range of mRNA targets. Furthermore, miRNAs are involved in all cellular activities, including biological and disease-related processes such as cell growth, division, differentiation, and apoptosis. Additionally, lncRNAs are a class of transcriptional regulators transcribed by RNA polymerase II, which are involved in alternative splicing and protein post-translational modification of mRNA. They competitively bind to miRNAs to regulate various miRNAs [66,67,68]. CircRNAs consist of introns, exons, or both and lack polyadenylated tails. The biogenetic pathways of circRNAs are varied, including but not limited to classical splicing and reverse splicing [69]. CircRNAs primarily act as miRNA sponges, protein bait antagonists, RNA splicing regulators, parental gene transcription regulators, and even translation templates to control the physiological and pathological functions of cells [70,71]. Some circRNAs have been found to function as molecular sponges to control the mRNA binding of proteins. Hence, circRNAs are likely more stable and suitable as biomarkers than lncRNAs [20].

### 3.2. The Role of ncRNAs in CIRI

ncRNAs do not encode proteins but are involved in various physiological and pathological processes, including cell pyroptosis [17], proliferation [72], and apoptosis [73]. Dysregulated ncRNA levels have been linked to a variety of diseases, including tumors, diabetes, and various types of ischemia-reperfusion injury [74,75]. ncRNAs, including miRNAs such as miR-155 [76], miR-377 [77], and miR-1202 [78], lncRNAs such as SNHG12 [79], MALAT1 [80], and ANRIL [81], and circRNAs such as circ-camk4 [82], circ0072309 [83], and circCCDC9 [84], are closely associated with CIRI. These can serve as biomarkers and therapeutic targets for neuroinflammation in ischemic stroke [21,85,86,87].

## 4. The Role of ncRNAs-Mediated Pyroptosis in CIRI

ncRNAs may contribute to CIRI by regulating the transcription or translation of pyroptosis-related genes. The role of ncRNAs-mediated pyroptosis in CIRI has garnered growing interest among researchers.

### 4.1. The Role of lncRNAs-Mediated Pyroptosis in CIRI

Recently, an increasing amount of research has documented the impact of lncRNA-regulated pyroptosis in CIRI.

X-inactive-specific transcript (XIST) is associated with abnormal inflammation regulators and acts as an sponge of miR-96-5p in CIRI [88]. In MCAO rats and OGD/R microvascular endothelial cells, XIST expression was downregulated, while NLRP3, Caspase-1, and GSDMD were upregulated, leading to increased IL-1β secretion. Protocatechol or MCC950 (pyroptosis inhibitor) improved cell damage and enhanced XIST expression. However, in the XIST silencing group, protocatechol did not alleviate pyroptosis in microvascular endothelial cells, thereby alleviating CIRI [47].

Opa-interacting protein 5 antisense transcript 1 (OIP5-AS1) is a highly conserved gene and plays an essential role in various diseases, including nervous system disorders, tumors, and inflammation [89]. A previous study has shown that overexpression of OIP5-AS1 in MCAO/R rats downregulates the expression levels of miR-186-5p, attenuates oxidative stress, and reduces the inflammatory response [90]. OIP5-AS1 was significantly downregulated in OGD/R neurons, MCAO/R mice, and ischemic stroke patients. Overexpression of OIP5-AS1 promoted thioredoxin-interacting protein (TXNIP) ubiquitination and degradation via the E3 protein ubiquitin ligase (E3) Itch, suppressed NLRP3 expression in brain tissue, inhibited neuronal pyroptosis, and alleviated CIRI [91].

Recent studies have documented that lncRNA Gm44206 has been closely linked to CIRI. OGD/R microglia showed enhanced the expression of Gm44206. In addition, NLRP3, Caspase-1, GSDMD, and apoptosis-associated speck-like proteins containing C-terminal caspase recruitment domains (ASC) were involved in microglial pyroptosis. Silencing lncRNA Gm44206 could reduce pro-inflammatory cytokine release, including IL-1 β, IL-6, IL-18, and TNF-α, through the classical pathway, thereby alleviating OGD/R microglial pyroptosis, thereby alleviating CIRI [15].

Some studies have shown that maternally expressed gene 3 (MEG3) binds competitively to miRNAs and participates in apoptosis, endoplasmic reticulum stress, oxidative stress, inflammation, epithelial-mesenchymal transition (EMT), and other processes [92]. LncRNA MEG3 exerts a range of pathological effects on neurons, astrocytes, microglia, and endothelial cells, showing potential for clinical application in the prevention and treatment of encephalopathy [93,94]. Knockdown of MEG3 inhibited caspase-1 signaling, decreased the levels of absent in melanoma 2 (AIM2), ASC, cleaved-caspase-1, and GSDMD-NT, and inhibited pyroptosis and inflammation in both MCAO rats and OGD/R-treated neurons, thereby alleviating CIRI [95].

KCNQ1 opposite strand/antisense transcript 1 (KCNQ1OT1) has been shown to play a key role in hypoxia through inflammation and oxidative stress [96]. In CIRI, KCNQ1OT1 acts as a sponge for miR-153-3p [97], miR-140-3P [98], miR-30b [99], and miR-9 [100]. KCNQ1OT1 exhibited robust expression in murine and in vitro neuronal cell models. Specifically, knockdown of KCNQ1OT1 or overexpression of miR-153-3p attenuated OGD/R-induced neuronal injury and regulated Foxo3 expression to inhibit pyroptosis, thereby alleviating CIRI [97].

Many studies have shown that lncRNA Taurine Up-regulated Gene 1 (TUG1) plays a key role in the I/R-related inflammatory response [101]. TUG1 downregulates miR-9a-5p and upregulates KLF5 expression, leading to cardiomyocyte apoptosis following myocardial ischemia and reperfusion [102]. The expression of TUG1 was upregulated in OGD/R-treated astrocytes, which in turn regulated miR-145 and aquaporin 4 (AQP4) to induce neuronal death [103]. Knockdown of TUG1 increased the level of miR-200a-3p, and decreased the levels of NLRP3, caspase-1, GSDMD-NT, IL-18,and IL-1β, thereby exacerbating CIRI [104].

Low levels of lncRNA RGD1564534 were found in MCAO rats and OGD/R cells. RGD1564534 could sponge miR-101a-3p to increase Dusp1 levels and upregulate its expression in neurons, thereby inhibiting the activation of the NLRP3 inflammasome. Its protective effect on neurons was related to promoting autophagy and inhibiting pyroptosis, thereby alleviating CIRI [105].

Recent studies have found that nuclear overexpressed transcript 1 (NEATl) is strongly associated with brain-related diseases. NEATl sponges miR-22-3p [106], miR-874-3p [107], and miR-214 [108], thereby exacerbating CIRI. In MCAO rats and OGD/R-treated neurons, NEAT1 was found to sponge miR-22-3p and decrease the expression of NLRP3 and cleaved caspase-1 to suppress pyroptosis, thereby alleviating CIRI [106].

Moreover, lncRNA metastasis-associated lung adenocarcinoma transcript 1 (MALAT1) was found to exacerbate cerebral infarction through the mouse double minute 2 (MDM2)-p53 pathway [109]. MALAT1 was highly expressed in a diabetic cerebral ischemia model, and MALAT1 knockdown of effectively inhibited inflammation and pyroptosis in BV2 cells. In addition, MALAT1 interacted with STAT1 and can enhance the transcriptional activity of NLRP3. Knockdown of STAT1 significantly inhibited MALAT1 transcription. Thus, the interaction between MALAT1 and STAT1 promoted diabetic cerebral ischemia-induced microglial pyroptosis by activating NLRP3 transcription, thereby alleviating CIRI [110].

LOC102555978 is a newly discovered CIRI-related lncRNA. LOC102555978 promoted NLRP3-mediated pyroptosis of microglia in MCAO/R rats and OGD/R microglia. Astragaloside IV inhibits microglia pyroptosis by downregulating LOC102555978, thereby alleviating CIRI [111].

Some studies have found that lncRNA FENDRR plays an important role in gastric cancer, osteosarcoma, bile duct cancer, and other diseases [112]. FENDRR can promote apoptosis in intracranial microvascular endothelial cells [113]. The results revealed increased expression of FENDRR, as well as upregulation of the NLRC4 inflammasome and pyroptosis in diabetic rats following CIRI. Similarly, in the OGD/R model, the expression of NLRC4 and related inflammatory factors decreased after FENDRR knockdown, thereby alleviating CIRI [114].

### 4.2. The Role of miRNAs-Mediated Pyroptosis in CIRI

Recently, miR-139 has been associated with inflammatory regulation in various diseases. Elevated miR-139 expression regulates the c-Jun/NLRP3 inflammasome pathway, thereby reducing CIRI [115]. In MCAO/R mice and OGD/R neurons, miR-139-5p was downregulated, the antioxidant pathway of Nrf2 was inhibited after the expression of the forkhead boxO1 (FoxO1) and Keap1 were up-regulated, and pyroptosis was mediated by NLRP3 inflammasome. Ginsenoside Rd inhibits the transcriptional regulation of Keap1 by FoxO1 by upregulating miR-139-5p, thereby activating the Nrf2 pathway and reducing pyroptosis mediated by the ROS/TXNIP/NLRP3 inflammasome pathway, thereby alleviating CIRI [116].

miR-155-5p is a newly discovered inflammatory regulator involved in a series of inflammatory diseases [117]. Previous studies have shown that miR-155-5p in CIRI can improve neuroinflammation by targeting dual-specificity phosphatase 14 (DUSP14) [118]. However, Que et al. established an elderly rat model and reported that the overexpression of DUSP14 could inhibit the NLRP3 inflammasome, reduce the inflammatory response, and improve cognitive function in elderly rats [119]. miR-155-5p, TXNIP, and NLRP3 were significantly upregulated in CIRI. In addition, knockdown of miR-155-5p inhibits inflammation and pyroptosis and plays a protective role in CIRI [120].

miR-96-5p plays a crucial role in cancer [121]. miR-96-5p lowered hypoxia-induced apoptosis and cardiac fibrosis in cardiomyocytes [122]. A previous study revealed that the expression of miR-96-5p is significantly downregulated in animal models of cerebral ischemia. miR-96-5p mimics significantly inhibited the expression of caspase-1 and improved pyroptosis in OGD/R treated N2a cells, while the miR-96-5p inhibitor caused the opposite results, thereby alleviating CIRI [123].

miR-29 is first discovered in human HeLa cells in 2001 and plays a critical role in cancer [124]. miR-29a-3p is associated with lung fibrosis [125] and cardiomyocyte apoptosis [126]. Astrocyte-derived extracellular vesicles (EVs) improved miR-29a expression in MCAO/R rat brains and OGD/R treatment N9 microglia. miR-29a from astrocyte-derived EVs alleviated CIRI by reducing tumor protein p53-Inducible Nuclear Protein 1 (TP53INP1) expression and the NF-κB/NLRP3 pathway to inhibit cell pyroptosis [127].

miR-135a-5p plays a pivotal role in numerous illnesses and is associated with autophagy, proliferation, apoptosis, etc. [128]. miR-135a-5P has been shown to prevent CIRI development [129]. Overexpression of miR-135a-5p was observed in EVs of M2 microglia, which delivered miR-135a-5p to neurons to inhibit TXNIP expression, thereby inhibiting NLRP3 inflammasome activation and reducing neuronal pyroptosis and cerebral ischemia [130].

miR-223 has been shown to be a biomarker for various human metabolic diseases and is involved in inflammation, autoimmune disorders, and other diseases. In addition, miR-223 protects against brain damage caused by glutamate excitotoxicity [131]. miR-223 contains a binding site in the 3′ UTR of NLRP3 mRNA, enabling its regulation [132]. Electroacupuncture was found to inhibit the increase of miR-223 expression in brain tissue after ischemia-reperfusion injury, while the expression of NLRP3, caspase-1, IL-1ß, and IL-18 decreased. However, antagomir-223 could partially inhibit the protective effect of electroacupuncture against cerebral ischemia [133].

miR-124 plays a vital neuroprotective role, and injection of miR-124 reduces neuronal damage induced by hypoxia and glucose deprivation in the early stages of ischemic stroke [134]. In addition, Ponomarev et al. showed that the higher levels of miR-124 prevented allergic meningitis by blocking microglial activation. The protective effect of miR-124 may be intricately linked to inflammation [135]. miRNA-124 agonists inhibited the expression and activation of STAT3 in a targeted manner, which also decreased the extent of pyroptosis. However, miR-124 antagonist reversed miR-124 agonist-mediated effects [136].

miR-423-5p is implicated in several diseases but has rarely been reported in CIRI. Luo et al. demonstrated that the levels of elevated miRNA-124 reduced I/R inflammation and pyroptosis in the brain through the NLRP3/caspase-1 pathway. Additionally, the knockdown of miR-423-5p improved venous nerve parameters such as cerebral infarct area, nerve score, cerebral edema, and neuronal injury, while inhibiting NLRP3 inflammasomes, pyroptosis, and oxidative stress [137].

### 4.3. The Role of circRNAs-Mediated Pyroptosis in CIRI

Compared with other ncRNAs, the circular structure of circRNAs provides higher stability for miRNA binding and plays a vital role in diagnosis. However, the mechanism of circRNA-mediated pyroptosis in CIRI remains poorly understood. CircRIMS is recognized as a proto-oncogenic circular RNA in gastric cancer and esophageal squamous cell carcinoma [138]. Li W et al. found that the levels of circRIMS were elevated in both the MCAO/R model and OGD/R model and that circRIMS contributed to CIRI progression through the regulation of the miR-96-5p/JAK/STAT1 axis [139]. Overexpression of miR-96-5p can inhibit pyroptosis and alleviate CIRI in mice [123].

CircCCDC6 is a recently discovered circRNA associated with CIRI. MCAO/R and OGD/R-treated SH-SY5Y cells showed increased levels of circCCDC6. Silencing circCCDC6 reduced neuronal pyroptosis and inflammation in MCAO/R rats. CircCCDC6 modulates miR-128-3p to trigger TXNIP/NLRP3, promoting pyroptosis and inflammation in response to OGD/R [140].

**Figure 3:**
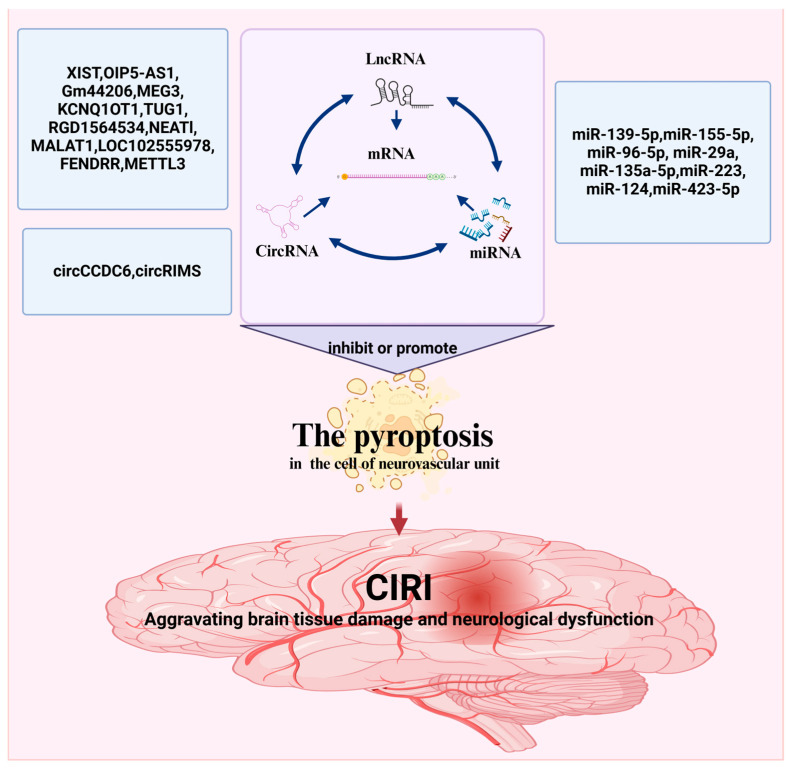
ncRNAs-mediated regulation of pyroptosis in CIRI. The figure was created using BioRender.

ncRNAs are widely present in various human organs. ncRNAs are released into the blood according to the degree of pyroptosis. As shown above, altering ncRNA expression levels in CIRI can promote pyroptosis (Figure 3 and Table 1).

## 5. Conclusions and Prospect

This narrative review, rather than a systematic review, did not follow a formal systematic review or scoping review protocol. We found that ncRNAs affected the pathological process of CIRI by influencing the pyroptosis of and cerebral microvascular endothelial cells, neurons, microglia and astrocytes, and other cells [136,141]. Pyroptosis-related ncRNAs form a complex regulatory network, providing new insights into the development and progression of CIRI and offering potential applications for early screening, diagnosis, and treatment. Extensive research has been conducted on pyroptosis-related lncRNAs and miRNAs, but the role of pyroptosis-associated circRNAs in CIRI remains largely unknown. Further research may uncover additional ncRNAs associated with pyroptosis, contributing to a better understanding of how CIRI works.

Currently, research is still in its early stages. Future studies should further explore the regulatory mechanisms of ncRNAs in CIRI, particularly the interactions between ncRNAs and pyroptosis, as well as functional differences in various types of cell. In terms of clinical applications, although ncRNAs has great potential as a therapeutic target, the development of its stability and delivery systems remains a pressing issue. Future research should focus on developing more effective ncRNAs delivery systems to enhance stability and targeting capabilities within the body. Additionally, combining ncRNAs-regulated pyroptosis strategies with other therapeutic approaches, such as pharmacological interventions and acupuncture, may yield better therapeutic outcomes. In summary, the role of ncRNAs in regulating pyroptosis provides new ideas and directions for treating CIRI, but further research and validation are necessary for clinical application.

## Figures and Tables

**Table 1:** The role of ncRNAs-mediated pyroptosis in CIRI.

ncRNAs	Class	Animals/Cells	Expression on Disease	Targets	Effect on Pyroptosis	Function
XIST	lncRNA	Rat/ BMECs	↓	NLRP3/Caspase-1/GSDMD	Anti-	Protocatechaldehyde attenuates rBMEC pyroptosis by inhibiting NLRP3/Caspase-1/GSDMD pathway through lncRNA XIST, thereby preventing ischemic injury [47].
OIP5-AS1	lncRNA	Rat/ Neurons	↓	ITCH/TXNIP	Anti-	OIP5-AS1 can negatively regulate TXNIP protein in vitro and in vivo, inhibit neuronal pyroptosis, and alleviate nerve damage after CIRI [89].
Gm44206	lncRNA	Microglia	↑	NLRP3/Caspase-1/GSDMD	pro-	Gm44206 promotes microglial pyroptosis and exacerbates CIRI via the classical pathway [15].
MEG3	lncRNA	Rat/Mice	↑	miR-485/AIM2	pro-	MEG3 enhances pyroptosis by targeting the miR-485/AIM2 axis to promote cerebral ischemia and reperfusion injury [95].
KCNQ1OT1	lncRNA	Mice/ Neurons	↑	MIR-153-3P/FOXO3	pro-	KCNQ1OT1 modulates the miR-153-3P/FOXO3 axis to increase pyroptosis, thereby facilitating oxygen loss and glucose/reoxygenation-induced neuronal damage [97].
TUG1	lncRNA	Mice/ Neurons	↑	miR-200a-3p/NLRP3	pro-	TUG1 sponges miR-200a-3p, reducing miR-200a-3p levels and promoting the NLRP3-dependent inflammatory response, therefore enhancing pyroptosis to promote ischemia and reperfusion [104].
RGD1564534	lncRNA	Rat/ Neurons	↓	miR-101a-3p/Dusp1/NLRP3	Anti-	RGD1564534 enhances mitophagy, reduces NLRP3 inflammasome activity, and inhibits OGD/R-induced neuronal pyroptosis [105].
NEAT1	lncRNA	Rat/ Neurons	↑	miR-22-3p/NLRP3	pro-	Gastrodin alleviates cerebral ischemia/reperfusion injury by managing the lncRNA NEAT1/miR-22-3p axis and inhibiting pyroptosis [106].
MALAT1	lncRNA	Mice/ Microglia	↑	STAT1/NLRP3	pro-	MALAT1 plays a role in the pathogenesis of brain I/R damage through the MALAT1/STAT1-mediated microglial pyroptosis [110].
LOC102555978	lncRNA	Rat/ Microglia	↑	miR-3584-5p/NLRP3	pro-	The effect of astragaloside IV on NLRP3 through LOC102555978 in reducing microglial pyroptosis induced by cerebral ischemia/reperfusion [111].
FENDRR	lncRNA	Rat/ Microglia	↑	HERC2/NLRC4	pro-	FENDRR can accelerate pyroptosis of microglia by inhibiting the ubiquitination and degradation of NLRC4 through E3 ubiquitin ligase [114].
miR-139	miRNA	Mice/ Neurons	↓	FoxO1/Keap1/Nrf2 or c-Jun/NLRP3	Anti-	Through negative regulation of c-Jun/NLRP3 inflammasome signaling, the upregulation of miR-139 provides protection for nerve damage caused by OGD/R [115].
miR-155-5p	miRNA	Rat/ Neurons	↑	DUSP14/TXNIP/NLRP3	pro-	Knockdown of miR-155-5p mediates brain inflammation by modulating the DUSP14/TXNIP/NLRP3 axis. These results could offer a potential approach for reducing brain ischemia-reperfusion damage [120].
miR-96-5p	miRNA	Mice/ Neurons	↓	caspase 1/GSDMD	Anti-	Inhibition of caspase-1 on the shielding effect of miR-96-5p against ischemic stroke in vitro and vivo [123].
miR-29a	miRNA	Rat/ Microglia	↓	TP53INP1 and the NF-κB/NLRP3	Anti-	Astrocyte-derived EVs containing miR-29a inhibit BIRI by downregulating TP53INP1 and NF-κB/NLRP3 [127].
miR-135a-5p	miRNA	Mice/ Neurons/Rat	↓	TXNIP/NLRP3 and mTOR/NLRP3	Anti-	In M2 microglia-derived EVs, increased expression of miR-135a-5p inhibits NLRP3 inflammasome via TXNIP downregulation, and inhibits neuronal pyroptosis [128,130].
miR-223	miRNA	Rat	↓	NLRP3/caspase-1	Anti-	miR-223 levels were reduced, whereas NLRP3, caspase-1, IL 1β, and IL-18 levels were elevated in the peripheral cerebral cortex of MCAO rats [133].
miR-124	miRNA	Rat	↓	STAT3/caspase-1	Anti-	During CIRI, miR-124 may provide neuroprotection against pyroptosis, possibly by inhibiting the signaling pathway of STAT3 [136].
miR-423-5p	miRNA	Mice	↑	NLRP3/caspase-1	pro-	The miR-423-5p inhibits the inflammation of I/R and cell pyroptosis in the brain through the NLRP3/caspase-1 pathway [137].
CircCRIM1	circRNA	Mice/Astrocytes	↑	miR-96-5p/JAK/STAT1	pro-	CircRIMS is involved in CIRI injury by regulation of the miR-96-5p/JAK/STAT1 axis [139].
CircCCDC6	circRNA	Rat/ Neurons	↑	microRNA-128-3p/TXNIP/NLRP3	pro-	CircCCDC6 mediates miR-128-3p and activates TXNIP/NLRP3, thereby facilitating neuronal pyroptosis and inflammation caused by OGD/R [140].

Note: In the table, the arrow ↑ indicates upregulation of the ncRNA in CIRI, while the arrow ↓ indicates downregulation of the ncRNA in CIRI.

## Data Availability

Data sharing is not applicable for this article, as no new data were developed or analyzed.

## Abbreviations

The following abbreviations are used in this manuscript:

KCNQ1OT1	KCNQ1 opposite strand/antisense transcript 1
IL-1β	interleukin-1β
IL-18	interleukin-18
GSDMD-NT	GSDMD-N-terminal fragments
PD-L1	programmed death-ligand 1
p-STAT3	phosphorylated STAT3
CLIC4	chloride intracellular channel 4
iNOS	inducible nitric oxide synthase
TNF-α	tumor necrosis factor-alpha
DGCR8	DiGeorge Critical Region 8
3′-UTR	3′-untranslated region
OIP5-AS1	Opa-interacting protein 5 antisense transcript 1
TXNIP	thioredoxin-interacting protein
MEG3	maternally expressed gene 3
EMT	epithelial-mesenchymal transition
AIM2	absent in melanoma 2
TUG1	Taurine Up-regulated Gene 1
MDM2	mouse double minute 2
FoxO1	forkhead boxO1
TP53INP1	tumor protein p53-Inducible Nuclear Protein 1
CIRI	Cerebral ischemia-reperfusion injury
ncRNAs	non-coding RNAs
caspase	cysteine aspartate-specific proteases
lncRNAs	long non-coding RNAs
miRNAs	microRNAs
circRNAs	circular RNAs
GSDM	gasdermin
NLRP3	NOD-like receptor thermal protein domain-associated protein 3
PRRs	pattern recognition receptors
OGD/R	oxygen-glucose deprivation/reoxygenation
MCAO/R	middle cerebral artery blockage/restoration
SDSS	Sodium Danshensu
BRCC3	BRCA1/BRCA2-Containing Complex Subunit 3
M1	the pro-inflammatory microglia
M2	the anti-inflammatory microglia
pre-miRNAs	Precursor microRNAs
TRBPs	TAR RNA-binding proteins
RISC	RNA-induced silencing complex
MREs	miRNA response elements
CSF	cerebrospinal fluid
XIST	X-inactive-specific transcript
ASC	apoptosis-associated speck-like proteins containing a C-terminal caspase recruitment domain
AQP4	encoding aquaporin 4
NEATl	nuclear abundance transcript 1
MALAT1	metastasis-associated lung adenocarcinoma transcript 1
DUSP14	Dual specificity ATPase 14
EVs	extracellular vesicles
